# Prevalence and correlates of diagnosed and undiagnosed diabetes mellitus among adults in rural Uganda during the COVID-19 pandemic: an evaluation of a community-based screening programme

**DOI:** 10.1093/inthealth/ihaf050

**Published:** 2025-05-02

**Authors:** Wenceslaus Sseguya, Silver Bahendeka, Nimesh Mody, Sara MacLennan, Aravinda M Guntupalli

**Affiliations:** Institute of Applied Health Sciences, University of Aberdeen, Aberdeen, AB24 3FX, UK; Department of Internal Medicine, St Francis Hospital Nsambya, P.O. Box 7146, Kampala, Uganda; Department of Internal Medicine, St Francis Hospital Nsambya, P.O. Box 7146, Kampala, Uganda; Department of Internal Medicine, Mother Kevin Postgraduate Medical School, Uganda Martyrs University, P.O. Box 5498, Kampala, Uganda; Institute of Medical Sciences, University of Aberdeen, Aberdeen, AB24 3FX, UK; Institute of Applied Health Sciences, University of Aberdeen, Aberdeen, AB24 3FX, UK; Institute of Applied Health Sciences, University of Aberdeen, Aberdeen, AB24 3FX, UK

**Keywords:** COVID-19 pandemic, diabetes mellitus, prevalence, rural, Uganda

## Abstract

**Background:**

One in two people with diabetes in sub-Saharan Africa remains undiagnosed, which has contributed to the region's high rates of diabetes-related morbidity and mortality. While the COVID-19 pandemic likely exacerbated diabetes prevalence, limited data from the region, including Uganda, hampers our understanding of this impact. To address this gap, we analysed the diabetes prevalence and correlates among adults from three rural districts of Uganda using data from a community-based screening programme conducted by community health workers (CHWs) during the pandemic.

**Methods:**

We used anonymised data of 2587 records to analyse the prevalence and correlates of diagnosed and undiagnosed diabetes. Prevalence was presented as proportions with 95% CIs. Correlates of diabetes and undiagnosed diabetes prevalence were analysed using logistic regression and presented as ORs.

**Results:**

The overall prevalence of diabetes was 11.3% (95% CI 10.2 to 12.5%), with a 7.2% (95% CI 6.2 to 8.1%) prevalence for diagnosed diabetes. A high proportion (36.4%) of diabetes cases were undiagnosed. Older age, high body mass index and pre-existing hypertension were associated with high diabetes prevalence.

**Conclusions:**

There was a high proportion of undiagnosed diabetes among rural adults during the pandemic. Engaging CHWs in routine diabetes screening and awareness programmes can reduce the burden of undiagnosed diabetes.

## Introduction

Diabetes affects an estimated 537million people globally and remains one of the leading causes of disability and mortality.^1^ Sub-Saharan Africa (SSA) bears the second largest disability and premature mortality burden of the disease.^2^ Also, in 2021, the region was projected to experience the highest increase in diabetes prevalence (129%) by 2045.^1,2^ Despite this alarming impact, the region still records the highest proportion of undiagnosed diabetes than any other, presenting a missed opportunity for early treatment initiation that would prevent or delay diabetes complications.^1^ Despite the healthcare burden and high treatment costs associated with delayed diagnosis, SSA countries, like Uganda, still grapple with challenges in implementing early diabetes screening due to limited funding, particularly in rural community settings.^3^

There have been concerns about the recent rise in diabetes prevalence exacerbated by the COVID-19 pandemic.^4,5^ This has been attributed to the direct link between COVID-19 and new-onset diabetes and the indirect COVID-19 pandemic measures that may have worsened metabolic risk factors for diabetes.^4,6^ Inadequate screening or inaccessibility to diagnostic services under such circumstances could limit timely diagnosis and management and thus worsen diabetes-related morbidity and mortality.^1^ The prevalence of diabetes during the COVID-19 pandemic has been reported across the world, with figures ranging from 6.89% in Greece to 18.10% in the USA.^7,8^ On the other hand, reported undiagnosed diabetes prevalence figures have ranged from 5.0% in Egypt to 8.6% in Ethiopia.^9–11^ In SSA, several approaches towards increasing diabetes screening have been reported, such as integrating screening into existing community-based health programmes. Kachimanga et al. showed that a community screening programme in Malawi, allowing those found with elevated blood pressure and or blood glucose levels to be referred for further clinical evaluation, increased screening uptake and the early detection of cases.^12^ Such approaches of screening individuals from their communities also reduce the overloading of health systems, which are already burdened by communicable diseases.

Uganda's COVID-19 pandemic lockdown measures were some of the longest in the region, ending with the reopening of schools after almost 2 y of COVID-19–related closure.^13,14^ No other country in SSA recorded any lockdown measures that lasted that long.^13^ Uganda has also recorded a growing trend in diabetes prevalence over the past decade, particularly among the rural population.^15,16^ National diabetes prevalence statistics published from the 2023 non-communicable disease (NCD) STEPS survey show a national prevalence rate of 2.6% (95% CI 2.0 to 3.3%) that is closely similar to the rural prevalence rate (2.6%; 95% CI 1.9 to 3.5%).^15^ The proportion of undiagnosed diabetes, as high as 49%, has also been reported in Uganda's general population before the pandemic.^16^ Uganda implements a decentralised healthcare system with a universal healthcare policy that grants all residents free access to healthcare services. However, healthcare services, including diabetes diagnostics and treatment, are immensely underfunded and under-resourced, and worsened during the pandemic, especially in rural settings.^17,18^

There is still a dearth of recent rural-specific evidence on overall diabetes prevalence and the proportion of undiagnosed diabetes in Uganda during the pandemic. To address this knowledge gap, we aimed to evaluate baseline data of a community diabetes screening programme conducted using village-based community health workers (CHWs) during the COVID-19 pandemic in three rural districts. This community screening programme was implemented to increase early detection and monitoring of diabetes and its risk factors. Unlike many small diabetes prevalence studies conducted in rural Uganda, this study presents recent findings in the context of the COVID-19 pandemic.

## Materials and methods

We used anonymised data from a screening baseline survey of an ongoing community project (WDF19-1721) titled ‘Integrated Non-Communicable Disease Management at Primary Health Care Level in Western Uganda’.^19^ The project was implemented in the rural districts of Sheema, Isingiro and Ntungamo by St Francis Hospital Nsambya in partnership with the Ministry of Health Uganda and the World Diabetes Foundation. We used the data to analyse the prevalence and correlates of diagnosed and undiagnosed diabetes among adults aged ≥30 y during the COVID-19 pandemic.

### Data sources and population

The data were collected by trained Village Health Teams (VHTs) from April 2021 to March 2022 from adult males and females aged ≥30 y. They captured individual characteristics, including age, sex and other sociodemographic, as well as clinical and anthropometric, parameters. For our study, data records of pregnant women were excluded from our analysis because diabetes in pregnancy was not considered in our scope of prevalence analysis.

### Settings

The three predominantly rural districts of Sheema, Isingiro and Ntungamo are adjacent to each other, bordering Rwanda and Tanzania as a three-district block. The districts have an estimated combined population of 1.4 million, whose primary source of livelihood is crop and animal farming.^19^ In Uganda, a typical village forms the lowest political administrative unit, usually consisting of 50–70 households, with a typical average population of 250–500 people and a household number of 45–150. Households can further be organised into community administrative units referred to as cells. Community healthcare programmes in each village are monitored and coordinated by a selected two-member volunteer (male and female) VHT, whom the Ministry of Health mandates to provide an extension of preventive and promotive healthcare services by linking with primary healthcare centres.^20^ The three districts are mainly served by government healthcare facilities at parish level, subcounty, subdistrict and district levels. Diabetes care services, including screening, are only available at healthcare facilities at the subcounty level or higher.

### Collection of data on variables

A pair of 40 VHTs per health facility-linked village, constituting 80 members, were trained to conduct screening for diabetes and capture sociodemographic and anthropometric data of adults aged ≥30 y from 1291 households in 40 villages. A team comprising two resident VHT members (one male and one female) was assigned to each village to conduct non-mandatory household-to-household screening organised in cell clusters of villages. Each team was allocated and provided with a blood glucose test kit (URIT-25, URIT Medical Electronic Co. Ltd) and test strips (URIT-G25, URIT Medical Electronic Co. Ltd), a digital weighing scale (Constant-A16998, Shahe Langge Electronic Products Co., Ltd), a non-stretch height measuring tape (COLT, SinoTools Industrial), a non-stretch waist measuring tape, a data entry book to record assessment parameters, a VHT screening reference handbook and personal protective supplies. The VHTs had undergone training under the project on interview skills and measurement of weight, height and waist circumference based on published anthropometric procedures.^21^ They assessed and captured participant sociodemographic parameters, weight (kg), height (cm), waist circumference (cm) and random capillary blood glucose (mmol/L). The data entry books were submitted for data entry into the project's electronic data registry. Individuals found with undiagnosed abnormal blood glucose were referred by the VHTs to the respective linkage health facilities for further clinical evaluation to diagnose and initiate diabetes treatment. A total of 73 of the 1291 households did not participate in the screening, representing a low proportion (5.7%) of non-participation. These included 11 households where adults were reported absent at the time of survey contact visits and 62 households whose occupying adults reportedly declined to take part. Because this was a non-mandatory survey, participants were not required to provide reasons for participation or non-participation.

### Measurement of blood glucose

The blood glucose test was performed with capillary blood drawn from a fingerprick. This measured random blood glucose. Although possessing limited diagnostic accuracy, random blood glucose has been documented as an appropriate method with which to identify potential cases that require further diagnostic evaluation because of its convenience, accessibility and increased uptake in population-based diabetes screening.^22,23^ Random blood glucose was therefore adopted in the screening survey to identify individuals with elevated blood glucose that require clinical intervention as a pilot for potential population-wide approaches to timely diabetes diagnosis.

### Definition of diabetes

Diagnosed diabetes was defined as participants' self-reported diagnosis of diabetes or receiving diabetes treatment.

Undiagnosed diabetes was defined as a random capillary blood glucose value of ≥11.1 mmol/L (200 mg/dl) in participants without a history of diabetes at the time of the survey.

Diabetes was defined as the total of diagnosed and undiagnosed cases in the study population.

### Covariates

Variables assessed included age (in years), sex (male/female), self-reported history of diabetes (yes/no), history of hypertension (yes/no), current alcohol use (yes/no), current smoking or tobacco use (yes/no), education level (no education, primary, secondary, tertiary) and housing status (poor, average, good). Physical activity was computed from self-reported activities and frequencies as sedentary (not engaged in any physical work), lightly active (engaged only in light home activities such as house sweeping, cleaning utensils, cooking, shop-keeping), moderately active (≤2 d of moderate-intensity physical work such as cattle grazing, farm clearing, pruning banana plantation) or very active (>2 d of high-intensity physical work such as digging, bricklaying, building, wood cutting, farm clearing).^24,25^ Body mass index (BMI) was computed as weight (kg) divided by the square of height (m) and categorised as underweight (<18.5 kg/m^2^), normal (18.5–24.9 kg/m^2^), overweight (25–29.9 kg/m^2^) and obese (≥30 kg/m^2^).^26^ Abdominal obesity was computed from waist-for-height ratio as waist (cm) divided by height (cm) and simply categorised as healthy central adiposity (<0.5) and high central adiposity (≥0.5).^26^

### Statistical analyses

We used SPSS version 28.0 (IBM Inc., Armonk, NY, USA) to analyse the prevalence and correlates of diabetes and undiagnosed diabetes. We summarised participant characteristics using numbers (counts) and proportions (%). The prevalence of undiagnosed diabetes in our study is the proportion of adults aged ≥30 y who were unaware of their elevated blood glucose levels (≥11.1 mmol/L) prior to the community screening survey. The prevalence of diabetes is the proportion of diagnosed and undiagnosed diabetes among all the adults aged ≥30 y screened during the survey. The prevalence rates are expressed as percentages (%) with 95% CIs. χ^2^ and Mann–Whitney U tests were used to test differences in categorical and continuous variable characteristics between groups as appropriate. We performed logistic regression to identify correlates of diabetes and undiagnosed diabetes, where we included variables that yielded p<0.25 at bivariate analysis and a priori variables reported in predictor models from high-quality studies as covariates.^27^ By including variables that yielded p<0.25 at bivariate analysis, we aimed to reduce the possibility of missing a variable that would have yielded a significant value and vice versa. This consideration has also been suggested by Bursac et al.^28^ and supported by Malhotra.^29^ ORs and 95% CIs were obtained. Key assumptions for our logistic model included independence of errors, absence of multicollinearity, a lack of strongly influential outliers and missing data being missing completely at random. p<0.05 was used to determine statistical significance.

## Results

### Characteristics of the study population

We analysed data of 2587 records from the 2880 records of the community survey dataset. The 293 excluded records (10.2%) had missing or invalid data on blood glucose values: our defining primary outcome. Participants included in the analysed records were mostly female (65.8%) and had a mean age and BMI of 50.4 (SD ±14.8) y and 25.2 (SD ±5.2) kg/m^2^, respectively. The majority of participants had completed primary-level education and were living in average housing conditions. Females had a higher mean BMI (23.8 vs 25.9 kg/m^2^, p<0.01) and higher waist circumference (p<0.001) than males, but a lower education level (p<0.01), tobacco smoking and alcohol use (p<0.001). Table 1 summarises the details of participant characteristics.

**Table 1.** Participant characteristics (n=2587 unless otherwise stated)

Characteristic	Overall	Men	Women	p
District, n (%)				NS^†^
Sheema	369 (14.3)	137 (15.5)	232 (13.6)	
Isingiro	1338 (51.7)	471 (53.3	867 (50.9)	
Ntungamo	880 (34.0)	276 (31.2)	604 (35.5)	
Age, mean (±SD), y	50.4 (14.8)	50.8 (14.5)	50.3 (15.0)	NS^§^
Age group, n (%), y				NS^†^
30–39	736 (28.4)	232 (26.3)	504 (29.6)	
40–49	589 (22.8)	200 (22.6)	389 (22.9)	
50–59	574 (22.2)	216 (24.4)	358 (21.0)	
60–69	351 (13.6)	129 (14.6)	222 (13.0)	
≥70	337 (13.0)	107 (12.1)	230 (13.5)	
BMI classification (n=2394), n (%), kg/m^2^				
Underweight (<18.5)	152 (6.3)	70 (8.7)	82 (5.2)	0.001^†^
Normal (18.5–24.9)	1141 (47.7)	460 (56.9)	681 (43.0)	
Overweight (25.0–29.9)	689 (28.8)	201 (24.8)	488 (30.8)	
Obese (≥30)	412 (17.2)	78 (9.6)	334 (21.0)	
Waist circumference (n=2401), n (%)^a^				*<*0.001^†^
Normal	888 (37.0)	587 (72.8)	301 (18.9)	
Abnormal	1513 (63.0)	219 (27.2)	1294 (81.1)	
Waist–height ratio (n=2002), n (%)				*<*0.001^†^
<0.5	475 (23.7)	239 (35.0)	236 (17.9)	
0.5–0.59	913 (45.6)	322 (47.1)	591 (44.8)	
≥0.6	614 (30.7)	122 (17.9)	492 (37.3)	
Physical activity, n (%)				
Very active	2584 (99.9)	883 (99.9)	1701 (99.9)	NS^†^
Moderately active	03 (0.1)	1 (0.1)	2 (0.1)	
Smoking and tobacco use, n (%)				*<*0.001^†^
Yes	229 (8.9)	110 (12.4)	119 (7.0)	
No	2358 (91.1)	774 (87.6)	1584 (93.0)	
Alcohol use, n (%)				*<*0.001^†^
Yes	596 (23.0)	335 (37.9)	261 (15.3)	
No	1991 (77.0)	549 (62.1)	1442 (84.7)	
Education level completed, n (%)				<0.001^†^
No formal education	474 (18.3)	80 (9.0)	394 (23.1)	
Primary education	1566 (60.6)	545 (61.7)	1021 (60.0)	
Secondary education	430 (16.6)	202 (22.9)	228 (13.4)	
Tertiary graduate	117 (4.5)	57 (6.4)	60 (3.5)	
Housing conditions, n (%)				NS^†^
Average	1826 (70.6)	663 (72.3)	1187 (69.7)	
Poor	761 (29.4)	245 (27.7)	516 (30.3)	

BMI: body mass index; IDF: International Diabetes Federation; NS: non-significant difference.

^a^IDF classification: normal (men: <94 cm; women: <80 cm), abnormal (men: ≥94 cm; women: ≥80 cm).

^†^χ^2^ test.

^§^Mann–Whitney U test.

### Prevalence of diabetes

**Figure 1.**
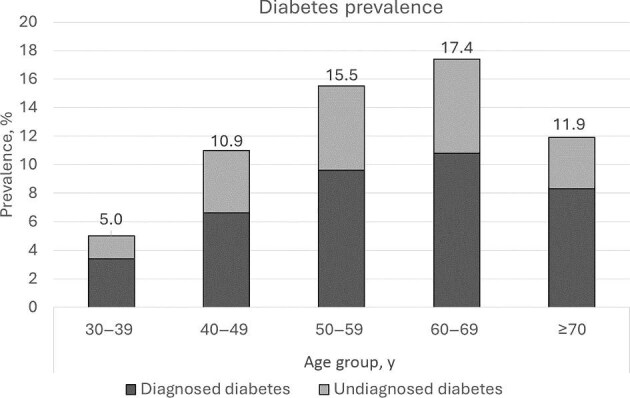
Age-specific prevalence rate of diabetes among adults in rural Uganda during the COVID-19 pandemic.

As shown in Table 2, the overall prevalence was 11.3% for diabetes (95% CI 10.2 to 12.5%), 7.2% for diagnosed diabetes (95% CI 6.2 to 8.1%) and 4.1% for undiagnosed diabetes (95% CI 3.4 to 4.9%). Additionally, the proportion of undiagnosed diabetes among diabetes cases was 36.4% (95% CI 30.9 to 42.0%). The prevalences of diagnosed and undiagnosed diabetes were highest among participants aged 60–69 y and lowest among those aged 30–39 y, as shown in Figure 1.

**Table 2.** Prevalence of diagnosed diabetes, undiagnosed diabetes and diabetes among adults in rural Uganda during the COVID-19 pandemic

			Undiagnosed diabetes		
	Total cases	Known diagnosed diabetes Prevalence, % (95% CI)	Prevalence, % (95% CI)	% of diabetes (95% CI)	Total diabetes Prevalence, % (95% CI)
Overall	291	7.2 (6.2 to 8.1)	4.1 (3.4 to 4.9)	36.4 (30.9 to 42.0)	11.3 (10.2 to 12.5)
Age group, y					
30–39	37	3.4 (2.1 to 4.7)	1.6 (0.7 to 2.5)	32.4 (16.6 to 48.3)	5.0 (3.4 to 6.6)
40–49	64	6.6 (4.6 to 8.6)	4.4 (2.5 to 6.1)	39.1 (26.8 to 51.3)	11.0 (8.3 to 13.4)
50–59	89	9.6 (7.2 to 12.0)	5.9 (4.0 to 7.9)	38.2 (27.9 to 48.5)	15.5 (12.5 to 18.5)
60–69	61	10.8 (7.6 to 14.1)	6.6 (4.0 to 9.2)	37.7 (25.2 to 50.2)	17.4 (13.4 to 21.4)
≥70	40	8.3 (5.3 to 11.3)	3.6 (1.6 to 5.5)	30.0 (15.2 to 44.8)	11.9 (8.4 to 15.3)
Sex					
Male	88	5.7 (4.1 to 7.2)	4.4 (3.1 to 5.8)	43.2 (32.6 to 53.7)	10.1 (8.0 to 11.9)
Female	203	7.9 (6.6 to 9.2)	4.0 (3.1 to 4.9)	33.5 (26.9 to 40.0)	11.9 (10.4 to 13.5)
District					
Sheema	32	6.0 (3.5 to 8.4)	2.7 (1.1 to 4.4)	31.3 (14.3 to 48.2)	8.7 (5.8 to 11.5)
Isingiro	175	7.9 (6.5 to 9.4)	5.2 (4.0 to 6.4)	39.4 (32.1 to 46.7)	13.1 (11.3 to 14.9)
Ntungamo	84	6.5 (4.8 to 8.1)	3.1 (1.9 to 4.2)	32.1 (21.9 to 42.3)	9.6 (7.6 to 11.6)
BMI category^a^, kg/m^2^					
Underweight	16	6.6 (2.6 to 10.6)	3.9 (0.8 to 7.1)	37.5 (10.9 to 64.1)	10.5 (5.6 to 15.5)
Normal	107	6.2 (4.8 to 7.6)	3.2 (2.2 to 4.3)	33.6 (24.5 to 42.7)	9.4 (7.7 to 11.1)
Overweight	89	7.5 (5.6 to 9.5)	5.4 (3.7 to 7.1)	41.6 (31.1 to 52.0)	12.9 (10.4 to 15.4)
Obese	59	9.5 (6.6 to 12.3)	4.9 (2.8 to 6.9)	33.9 (21.5 to 46.3)	14.3 (10.9 to 17.7)
Abdominal obesity^b^ (waist-for-height ratio)					
Normal (<0.5)	46	6.4 (4.3 to 8.7)	3.1 (1.6 to 4.7)	32.6 (18.5 to 46.7)	9.5 (6.9 to 12.2)
Abnormal (≥0.5)	190	7.6 (6.3 to 8.9)	4.0 (3.0 to 4.9)	34.7 (28.0 to 41.3)	11.6 (10.0 to 13.1)
Known hypertension					
No	150	4.7 (3.8 to 5.6)	2.5 (1.9 to 3.2)	34.7 (27.0 to 42.4)	7.2 (6.1 to 8.3)
Yes	141	17.7 (14.3 to 21.1)	11.0 (8.2 to 13.7)	38.3 (30.2 to 46.4)	28.7 (24.6 to 32.7)
Tobacco use					
No	265	7.2 (6.1 to 8.2)	4.1 (3.3 to 4.9)	36.2 (30.4 to 42.1)	11.3 (10.0 to 12.5)
Yes	26	7.0 (3.7 to 10.3)	4.4 (1.7 to 7.0)	38.5 (18.4 to 58.5)	11.4 (7.2 to 15.5)
Alcohol use					
No	219	7.3 (6.2 to 8.5)	3.7 (2.9 to 4.5)	33.3 (27.0 to 39.6)	11.0 (9.6 to 12.4)
Yes	72	6.5 (4.6 to 8.5)	5.5 (3.7 to 7.4)	45.8 (34.0 to 57.6)	12.0 (9.5 to 14.7)
Education level completed					
None	57	7.2 (4.8 to 9.5)	5.1 (3.1 to 7.0)	40.4 (27.2 to 53.5)	12.3 (9.1 to 15.0)
Primary	187	7.9 (6.6 to 9.3)	4.0 (3.0 to 5.0)	33.7 (26.9 to 40.5)	11.9 (10.3 to 13.5)
Secondary	37	5.3 (3.2 to 7.5)	3.3 (1.6 to 4.9)	37.8 (21.4 to 54.2)	8.6 (5.9 to 11.3)
Tertiary	10	3.4 (0.0 to 6.8)	5.1 (1.1 to 9.2)	60.0 (23.1 to 96.9)	8.5 (3.4 to 13.7)

BMI: body mass index.

a BMI classification computation had 193 (7.5%) missing values in the entire sample.

b Waist-for-height classification had 462 (17.9%) missing values in the entire sample.

### Correlates of diagnosed and undiagnosed diabetes

Factors associated with diabetes and undiagnosed diabetes are presented in Table 3. At bivariate logistic regression analysis, age, history of hypertension and BMI were found to be associated with diabetes and undiagnosed diabetes. Multivariate logistic regression analysis showed that the odds of having diabetes in participants aged 30–69 y significantly (p<0.019) increased with advancing age. On the other hand, the odds of having undiagnosed diabetes were only significantly (p<0.03) higher in the 50–69 y age range compared with the youngest age group (30–39 y). Additionally, having a history of hypertension was associated with higher odds of diabetes (adjusted OR [AOR] 5.4; 95% CI 4.0 to 7.3, p<0.001) and undiagnosed diabetes prevalence (AOR 5.2, 95% CI 3.2 to 8.4, p<0.001). Being overweight or obese was associated with 70% increased odds of having diabetes compared with individuals with normal BMI. On the other hand, compared with those with normal BMI, individuals who were overweight had 1.9 times higher odds of having undiagnosed diabetes. There were no associations at both bivariate and multivariate logistic regression analysis between diabetes and documented risk factors, namely, alcohol intake, tobacco smoking, education, level, sex and abdominal obesity.

**Table 3.** Factors associated with diabetes and undiagnosed diabetes among adults in rural Uganda during the COVID-19 pandemic

	Diabetes prevalence				Undiagnosed diabetes prevalence			
Factor Category	OR (95% CI)	p	AOR (95% CI)	p	OR (95% CI)	p	AOR (95% CI)	p
Age group, y								
30–39	1.0	Ref	1.0	Ref	1.0	Ref	1.0	Ref
40–49	2.3 (1.5 to 3.5)	**<**0.001	1.8 (1.1 to 2.9)	0.018	2.8 (1.4 to 5.6)	0.004	1.7 (0.7 to 3.9)	NS
50–59	3.5 (2.3 to 5.2)	<0.001	2.6 (1.6 to 4.1)	<0.001	3.8 (1.9 to 7.4)	<0.001	2.7 (1.3 to 5.9)	0.009
60–69	4.0 (2.6 to 6.1)	<0.001	2.7 (1.6 to 4.5)	<0.001	4.2 (2.1 to 8.6)	<0.001	2.5 (1.1 to 5.9)	0.029
≥70	2.5 (1.6 to 4.1)	<0.001	1.7 (1.0 to 3.0)	NS	2.2 (1.0 to 5.0)	NS	1.3 (0.5 to 3.5)	NS
Sex								
Male	1.0	Ref	1.0	Ref	1.0	Ref	1.0	Ref
Female	1.2 (0.9 to 1.6)	NS	1.2 (0.9 to 1.7)	NS	0.9 (0.6 to 1.3)	NS	0.9 (0.5 to 1.5)	NS
Known hypertension								
No	1.0	Ref	1.0	Ref	1.0	Ref	1.0	Ref
Yes	5.2 (4.0 to 6.7)	**<**0.001	5.4 (4.0 to 7.3)	**<**0.001	4.8 (3.2 to 7.0)	**<**0.001	5.2 (3.2 to 8.4)	**<**0.001
BMI, kg/m^2^								
Normal (18.5–24.9)	1.0	Ref	1.0	Ref	1.0	Ref	1.0	Ref
Underweight (<18.5)	1.1 (0.7 to 2.0)	NS	1.3 (0.7 to 2.5)	NS	1.2 (0.5 to 3.0)	NS	1.1 (0.4 to 3.3)	NS
Overweight (25.0–29.9)	1.4 (1.1 to 1.9)	0.018	1.7 (1.2 to 2.4)	0.006	1.7 (1.1 to 2.7)	0.027	1.9 (1.1 to 3.3)	0.028
Obese (≥30)	1.6 (1.1–2.3)	0.006	1.7 (1.1 to 2.7)	0.010	1.5 (0.9 to 2.7)	NS	1.7 (0.9 to 3.2)	NS
Abdominal obesity (waist-for-height ratio)								
Normal (<0.5)	1.0	Ref	1.0	Ref	1.0	Ref	1.0	Ref
Abnormal (≥0.5)	1.2 (0.9 to 1.7)	NS	0.9 (0.6 to 1.3)	NS	1.3 (0.7 to 2.3)	NS	1.0 (0.5 to 1.9)	NS
Current tobacco use								
No	1.0	Ref	1.0	Ref	1.0	Ref	1.0	Ref
Yes	1.0 (0.7 to 1.6)	NS	1.2 (0.7 to 2.1)	NS	1.1 (0.5 to 2.1)	NS	1.0 (0.4 to 2.3)	NS
Current alcohol use								
No	1.0	Ref	1.0	Ref	1.0	Ref	1.0	Ref
Yes	1.1 (0.8 to 1.5)	NS	1.0 (0.7 to 1.3)	NS	1.5 (1.0 to 2.3)	NS	1.5 (0.9 to 2.6)	NS
Education level								
None	1.0	Ref	1.0	Ref	1.0	Ref	1.0	Ref
Primary	1.0 (0.7 to 1.4)	NS	1.2 (0.8 to 1.7)	NS	0.8 (0.5 to 1.3)	NS	0.9 (0.5 to 1.6)	NS
Secondary	0.7 (0.4 to 1.1)	NS	1.4 (0.8 to 2.4)	NS	0.6 (0.3 to 1.2)	NS	1.1 (0.5 to 2.6)	NS
Tertiary	0.7 (0.3 to 1.4)	NS	0.6 (0.2 to 1.5)	NS	1.0 (0.4 to 2.5)	NS	0.9 (0.3 to 3.0)	NS

AOR: adjusted OR; BMI: body mass index; NS: non-significant difference.

AOR adjusted for age, sex, known hypertension, BMI, abdominal obesity, tobacco use, alcohol use and education level.

## Discussion

### Main findings

Our study aimed to report the prevalence and correlates of diabetes among adults aged ≥30 y during the COVID-19 pandemic from a community screening programme delivered using VHTs in rural Uganda. To the best of our knowledge, this is the first study in Uganda to report rural diabetes prevalence during the COVID-19 pandemic. Our findings highlight that CHWs can play a pivotal role in promoting early screening and identifying diabetes and its risk factors in rural settings without burdening the secondary and tertiary hospitals.

During the COVID-19 pandemic, we observed a high prevalence of diabetes in rural Uganda at 11%, with 36% of cases being undiagnosed. Diagnosed and undiagnosed diabetes cases were prevalent in 7% and 4% of adults, respectively. Being older, having existing hypertension and having a high BMI were independently significantly associated with diabetes.

### Diabetes prevalence among adults in rural Uganda during the COVID-19 pandemic

There was a high prevalence of diabetes among adults in rural Uganda during the COVID-19 pandemic. This rural diabetes prevalence is higher than the national rural prevalence figure (2.6%) reported in 2023,^15^ but lower than reported earlier (16.1%) in a rural district of southwestern Uganda.^30^ The figure is also significantly higher than in other rural parts of SSA.^31^ Various factors, including differences in study designs such as sample selection, study population characteristics, age characteristics and study time periods, may explain this variation. This high prevalence of diabetes observed among adults in rural Uganda during the COVID-19 pandemic may be explained by the documented COVID-19 pandemic exacerbation of diabetes incidence.^4,6^ The COVID-19 pandemic has been reported to have been associated with increased diabetes prevalence due to severe acute respiratory syndrome coronavirus 2 (SARS-CoV-2) infection-induced diabetes incidence and the pandemic's lockdowns aggravating metabolic and behavioural risk factors for diabetes.^6,32^ Uganda, like many countries in SSA, registered high rates of SARS-CoV-2 infection, which were not only experienced in urban but in rural areas as well.^33^ The country also imposed one of the longest COVID-19 lockdowns in the region, whose restrictions may have worsened the population risk factors for diabetes.^13^ The lockdown measures imposed in Uganda were largely similar to those in many countries within SSA, but some extended beyond what was seen in other countries within the region.^13,34^

### Undiagnosed diabetes among adults in rural Uganda during the COVID-19 pandemic

During the COVID-19 pandemic, 36% of diabetes cases in rural Uganda remained undiagnosed. This proportion is lower than reported in the general population of Uganda (49%) and SSA (53.6%).^1^ This may have been due to the increased media propagation of the adverse effects of COVID-19 in people with diabetes and the country's prioritisation of COVID-19 vaccination for people with diabetes, which may have been motivators for individuals to seek diagnostic services for diabetes.^18,35^ Nevertheless, the observed proportion of undiagnosed diabetes remains high and is of public health concern. This could be explained by the population's poor health-seeking and or the ineffectiveness of the healthcare system screening programmes in these rural settings.^36,37^ Undiagnosed diabetes has been shown to be associated with high disability-adjusted life years among people with diabetes, because many present late with irreversible complications.^2^ Additionally, it increases the diabetes treatment cost burden, which weighs down on individuals and the healthcare system. Uganda's healthcare system, like many countries in SSA, is considerably underfunded and still struggles with high out-of-pocket expenditure, especially for NCDs.^38^ Given the close coincidence of the age group with the highest number of cases of undiagnosed diabetes (50–69 y), the country's retirement age (60 y) and life expectancy (66 y), the threat of undiagnosed diabetes has grave economic implications.^39^

### Risk factors for diagnosed and undiagnosed diabetes during the pandemic

The diabetes risk factors—older age, high BMI and pre-existing hypertension—observed among adults in rural Uganda have also been reported elsewhere, and their causal relationship elaborated.^40^ Unlike many studies that have documented an association between diabetes and sex, abdominal obesity, tobacco smoking, alcohol use and education level, we did not observe any association of these among adults in rural Uganda. The Uganda national survey, from which population our study drew the sample, also reported no differences in diabetes prevalence between males and females.^15^ The lack of association between abdominal obesity and diabetes could be explained by the influence of ethnicity modulation of phenotypic characteristics that have been recently documented in African populations.^41,42^ However, it is important to note from this non-mandatory screening programme that a significantly higher number of women than men enrolled, yet were drawn from a population with a similar gender distribution ratio.^19^ Whereas poor health-seeking behaviour among men reported elsewhere may be attributed to this,^43^ there is a need for specific investigation into factors that may be associated with this variation in these rural settings. Such gender disparities in healthcare service uptake need to be adequately understood and considered to achieve effective coverage of diabetes screening programmes.

Our findings further suggested that undiagnosed diabetes was significantly higher in older adults, with a significant number among overweight but not obese adults. These results align with the theory suggesting that Africans may have a different diabetes phenotype, which commonly manifests among non-obese Africans.^41^ This may also be an underlying reason why the majority of the population without obvious risk factors, such as obesity, may be reluctant to screen. Screening programmes should, therefore, be sensitive enough to reach even those individuals with unconventional risk factors. Owing to the diversity and size of our study sample, our findings and implications are largely generalisable to rural Uganda and rural low-income countries with similar health system and population characteristics. Regions in Uganda with differences in dietary, anthropometric and socioeconomic characteristics, such as the north and northeastern, may exhibit different risk factors from those reported in our study.^19^

### Strength and limitations

The key strength of our study is that it used data within the COVID-19 context that provide a prevalence picture that is representative of COVID-19’s impact on the diabetes burden. Second, the study's data are drawn from three districts, which enhances the generalisability of findings. However, our study was limited by the fact that the assessment of undiagnosed diabetes was based on a single random capillary blood glucose measure, which may provide a clinically biased assessment. The use of fasting blood glucose or glycated haemoglobin would have provided a better assessment of diabetes. Nevertheless, random blood glucose has been reported to be highly sensitive and specific in facilitating the detection of diabetes.^22^

### Conclusions

There was a high prevalence of diabetes and a high proportion of undiagnosed cases among adults in rural Uganda during the COVID-19 pandemic. People who were older, overweight or obese, and those with pre-existing hypertension, were at an increased risk of developing diabetes. There is a need to increase access to screening and awareness of risk factors for diabetes among rural communities for early prevention of complications and premature disease mortality and disability. Diabetes screening and awareness programmes can be incorporated into existing community health structures fronted through empowering and building the capacity of CHWs. This can potentially increase sustainability and community uptake of diabetes screening programmes without overloading the already burdened secondary and tertiary healthcare facilities. However, for this to be feasible, more research to determine how such services can be effectively and contextually integrated into the healthcare system as a whole is needed.

## Data Availability

The data underlying this article were provided by St Francis Hospital Nsambya under permission. Data will be shared on request to the corresponding author with permission of St. Francis Hospital Nsambya.
